# Association between neutrophil-to-lymphocyte ratio and all-cause and cardiovascular mortality among adults with cancer from NHANES 2005-2018: a retrospective cohort study

**DOI:** 10.3389/fonc.2025.1521099

**Published:** 2025-03-18

**Authors:** Gangping Li, Yuewen Fu, Di Zhang

**Affiliations:** ^1^ Department of Hematology, The Affiliated Cancer Hospital of Zhengzhou University & Henan Cancer Hospital, Zhengzhou, China; ^2^ Department of Medical Records Management, The Affiliated Cancer Hospital of Zhengzhou University & Henan Cancer Hospital, Zhengzhou, China

**Keywords:** neutrophil, lymphocyte, all-cause mortality, cardiovascular disease, NHANES

## Abstract

**Background:**

Evidence on the association between the neutrophil-to-lymphocyte ratio (NLR) and all-cause and cardiovascular disease (CVD) mortality in adults with cancer is limited.

**Aims:**

This study aimed to examine the relationship between NLR and all-cause and CVD mortality in adults with cancer.

**Methods:**

A retrospective cohort study included 2,639 cancer patients in the U.S. from the NHANES dataset (2005-2018), collecting demographic, laboratory, and mortality data. Multivariable Cox regression analysis, subgroup analysis and restricted cubic spline analyses assessed the associations between NLR and mortality outcomes.

**Results:**

During a median follow-up of 77 months, 713 (27.0%) deaths occurred, including 149 (5.6%) from CVD. Multivariable Cox regression analysis revealed that a high NLR, treated as a continuous variable, was significantly correlated with increased all-cause mortality (HR, 1.09; 95% CI, 1.05-1.12; p < 0.001) and CVD mortality (HR, 1.12; 95% CI, 1.05-1.19; p < 0.001). Meanwhile, when evaluating NLR as a categorical variable, the adjusted hazard ratios (HR) for NLR and all-cause mortality in quartiles Q2 (1.6-2.2), Q3 (2.2-3), and Q4 (>3) were 1.06 (95% CI: 0.83-1.34, p = 0.062), 1.12 (95% CI: 0.89-1.42, p = 0.334), and 1.30 (95% CI: 1.04-1.63, p = 0.021), respectively, when compared with individuals in the lower quartile Q1 (≤1.6). In terms of CVD mortality, the adjusted HR values for NLR in Q2, Q3, and Q4 were 0.92 (95% CI: 0.50-1.69, p = 0.062), 1.24 (95% CI: 0.71-12.19, p = 0.334), and 1.76 (95% CI: 1.04-2.97, p = 0.034), respectively, compared to those in the lower NLR quartile Q1 (≤1.6). Subgroup analysis showed similar patterns (all p-values for interaction > 0.05). Kaplan-Meier analysis indicated lower survival rates for individuals with higher NLR, and RCS analysis suggested a positive linear relationship between NLR and all-cause and CVD mortality.

**Conclusion:**

Elevated NLR is linked to higher all-cause and CVD mortality risks among adults with cancer.

## Introduction

Cancer is a significant challenge in the 21st century, accounting for a large proportion of non-communicable disease-related deaths worldwide (1–3). It is a leading cause of premature mortality, particularly among individuals aged 30-69, and is among the top three causes of death in this age group in most countries (1). Cardiovascular disease (CVD) and cancer are the leading causes of death in 127 countries (4). Globally, there were approximately 19.3 million new cancer cases in 2022, with an incidence rate of 196.9 per 100,000 population (1). In 2020, about 10.6 million new cases of ischemic heart disease (IHD) were reported worldwide (5, 6). The incidence of IHD is higher among cancer patients, particularly in the elderly, due to shared risk factors and adverse effects of certain cancer treatments (7). Although cancer is a serious health concern, there is relatively less research on the overall mortality rate and cardiovascular mortality rate of patients with cancer than on cardiovascular diseases. CVD have long been among the leading causes of death worldwide, and research related to cancer has not received equal attention (8). This indicates the need for further research to gain a deeper understanding of the all-cause mortality rate in cancer patients and its association with the CVD mortality rate to better comprehend and address the challenges faced by cancer patients.

In the 19th century, Rudolf Virchow first noticed the presence of leukocytes in tumors and suggested that inflammation may play a role in promoting the growth of cancer cells (9). This discovery led to the recognition of inflammation as a key factor in cancer development, progression, and spreading (10, 11). Inflammation is considered a fundamental characteristic of cancer and is closely linked to various stages of the disease from its onset to the formation of metastases (12). There is growing interest in simple blood methods, such as the Neutrophil-to-Lymphocyte Ratio (NLR), to predict cancer prognosis and assess inflammatory conditions (13). NLR, obtained from complete blood counts, is an indicator of inflammation and a well-studied marker of survival in patients with cancer and cardiovascular disease (9, 14, 15). However, whether it is also predictive of all-cause and CVD mortality in cancer patients remains unknown. This study aimed to investigate the association between NLR and all-cause and CVD mortality in adults with cancer.

## Materials and methods

### Study design

**Figure 1:**
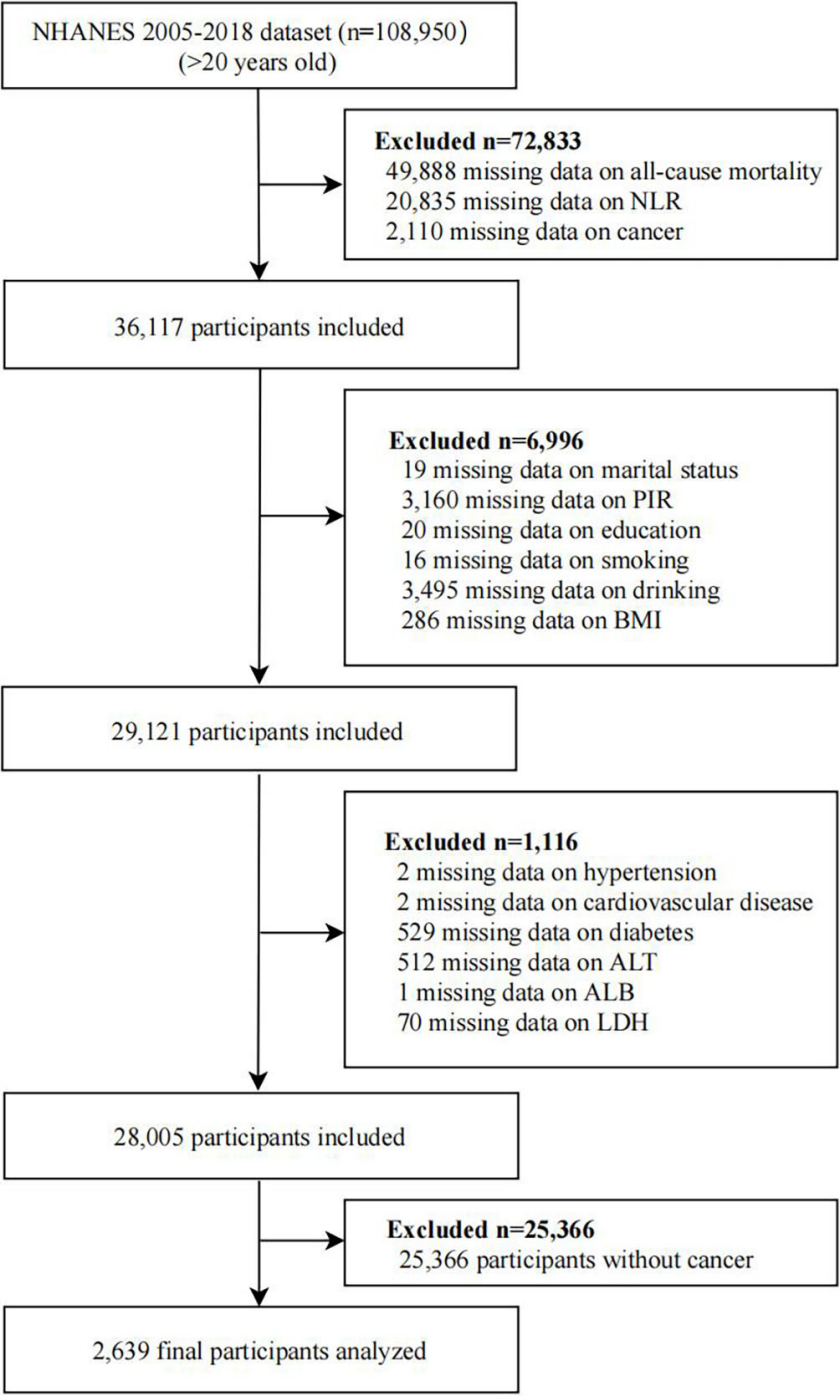
The flow chart of the study.

This retrospective cohort study utilized data from the National Health and Nutrition Examination Survey (NHANES) conducted by the Centers for Disease Control and Prevention between 2005 and 2018. The NHANES aims to assess the health and nutritional status of non-institutionalized Americans through a comprehensive survey using a stratified multistage probability sampling method. Data collection included demographic information, detailed health assessments, and laboratory tests performed at a mobile examination center (MEC) or through home visits. This study adhered to the principles outlined in the Declaration of Helsinki and was approved by the National Center for Health Statistics (NCHS) Research Ethics Review Board. All adult participants provided written informed consent before participation. Our secondary analysis adhered to the STROBE guidelines for cohort studies and did not require further approval from our institutional review board. The NHANES data used in this study are available to the public on the NHANES website. More information on NHANES data can be found on the website. (https://www.cdc.gov/nchs/nhanes/?CDC_AAref_Val=https://www.cdc.gov/nchs/nhanes/index.htm) (accessed March 1, 2022). Participants with a history of cancer or malignancy were identified based on their responses to the question, “Have you ever been told by a doctor or other health professional that you had cancer or a malignancy of any kind?” Participants who did not have complete data on risk behaviors, associated comorbid conditions, or demographic details were excluded from the study. The exclusion criteria are specified in **Figure 1**. In total, the analysis included 2,639 adults with cancer, and the specific types and frequencies of cancers are presented in **Supplementary Table S1**.

### Determination of mortality and follow−up

The determination of mortality status and follow-up involved linking the NHANES data with records from the National Death Index (NDI), which is accessible at https://www.cdc.gov/nchs/data-linkage/mortality-public.htm. Using this information, the participants were categorized as deceased or alive. The follow-up period was determined by measuring the time from the date of the NHANES examination to the date of death or December 31, 2019, whichever occurred first. The underlying causes of death were identified using the International Classification of Diseases, Tenth Revision (ICD-10), with a specific focus on cardiovascular mortality, which was classified by the NCHS as death attributed to heart disease based on ICD-10 codes I00-I09, I11, I13, and I20-I51 (16).

### Measurement of NLR

Neutrophil and lymphocyte counts were obtained from complete blood count analyses of blood samples using a Beckman Coulter automated blood analyzer at a mobile examination center (MEC), and the counts were expressed as × 10^3^ cells/µL. NLR was calculated by dividing the absolute neutrophil count by the absolute lymphocyte count.

### Covariates

Various potential covariates were considered in accordance with the existing literature. These included age, sex, race/ethnicity, marital status, education level, family income (PIR), body mass index (BMI), smoking status, hypertension, diabetes, CVD, and laboratory parameters such as hemoglobin, platelet count, alanine aminotransferase (ALT), creatinine, albumin, and lactate dehydrogenase levels (LDH). The participants were grouped into the following categories according to race/ethnicity: Non-Hispanic White, Non-Hispanic Black, Mexican American, and other races. The respondents’ marital status was classified as married, living with a partner, or living alone. Education level was classified as less than 9 years, 9–12 years, and > 12 years of education (13). Family income was categorized into low (poverty income ratio, PIR ≤ 1.3), medium (PIR > 1.3 to 3.5), and high (PIR > 3.5) based on a US government report (17). Smoking status was categorized as follows: never smokers (those who had smoked fewer than 100 cigarettes), current smokers, and former smokers (those who quit after smoking more than 100 cigarettes), following definitions from the literature (17). Participants were segmented based on their alcohol consumption patterns, with categories including never drinkers (< 12 drinks in their lifetime), former drinkers (≥ 12 drinks in 1 year but did not drink last year, or did not drink last year but consumed ≥ 12 drinks in their lifetime), current mild alcohol users (≤ 1 drink per day for females, ≤ 2 drinks per day for males), current moderate alcohol users (≥ 2 drinks per day for females, ≥ 3 drinks per day for males, or binge drinking ≥ 2 days per month), and current heavy alcohol users (≥ 3 drinks per day for females, ≥ 4 drinks per day for males, or binge drinking ≥ 4 drinks on the same occasion for females, ≥ 5 drinks on the same occasion for males on 5 or more days per month) (13). The presence of previous diseases such as hypertension, diabetes, and CVD was determined based on the participants’ responses to questions in the questionnaire regarding whether a doctor had diagnosed them with the condition in the past. BMI was calculated using a standardized technique that incorporates weight and height measurements.

### Statistical analysis

For normally distributed continuous variables, the mean and standard deviation (SD) were reported, while skewed continuous variables were described using the median and interquartile range (IQR). Categorical variables were presented as frequencies and percentages (%). To compare continuous variables among groups, the independent samples Student’s t-test or Mann-Whitney U-test was employed based on the normality of the distribution. Categorical data were compared using the chi-square test or Fisher’s exact test, as appropriate.

Multivariable Cox proportional hazards regression models were employed to assess the hazard ratio (HR) and 95% confidence interval (95% CI) for the relationship between NLR and the risks of all-cause and cardiovascular mortality. The NLR was entered as a categorical variable (four quartiles). We selected these confounders based on their judgments. We constructed three models. Model 1 was adjusted for age, sex, race, marital status, PIR, and education. Model 2 was additionally adjusted for Model1 and smoke, alcohol drinking status, BMI, hypertension, diabetes, and CVD, while Model 3 was additionally adjusted for Model2 and hemoglobin, platelet, ALT, creatinine, albumin, and LDH.

Tests for trends were conducted using multivariate regression models by entering the four quartiles of NLR as a categorical variable in the models. We used a restricted cubic spline model to develop smooth curves and examine the possible nonlinear dose-response associations between NLR and cancer. Nonlinearity was assessed using a likelihood ratio test, comparing the model with only a linear term against the model with linear and cubic spline terms. In the case of non-linear correlation, a two-piecewise regression model was applied to determine the threshold effect of the NLR on cancer, and this was illustrated using a smoothing plot. Subgroup analyses were also performed. For the continuous variable, we first converted it to a categorical variable according to four quartiles and then performed an interaction test. Missing data accounted for less than 5% of the dataset and were handled by listwise deletions on an analysis basis. We performed a series of sensitivity analyses to assess the robustness of the study’s findings and evaluate how our conclusions might be influenced by employing different association inference models. Formal interaction tests were performed using likelihood ratio tests. For multiple comparisons, we applied the Bonferroni correction and divided the analysis into five subgroups. The p-value for the interaction test was set at less than 0.01 (0.05/5), which we considered indicative of a statistically significant difference. Otherwise, no significant difference was assumed. We report and compare the effect sizes and p-values calculated using all these models. All analyses were conducted using R Statistical Software (version 4.2.2) and the Free Statistics Analysis Platform (version 1.9, Beijing, China; http://www.clinicalscientists.cn/freestatistics). Statistical significance was set at p < 0.05.

## Results

### Baseline characteristics

This study included 2,639 eligible aged 65.3 ± 14.1 years. During a median follow-up of 77.0 (45.0, 121.0) months, 713 deaths occurred, including 149 cardiovascular deaths. **Table 1** shows the general characteristics of the participants according to the NLR. The four groups differed in age, sex, race, PIR, smoking, alcohol consumption, hypertension, diabetes, CVD, platelet count, ALT, albumin, creatinine, and LDH (all p < 0.05).

**Table 1:** Population characteristics by categories of the NLR.

Characteristic	Neutrophil-lymphocyte ratio					
	Total	Q1 (≤1.6)	Q2 (1.6-2.2)	Q3 (2.2-3)	Q4(>3)	
No.	2639	656	663	630	690	*p*-Value
Age (year), Mean (SD)	65.3 ± 14.1	62.2 ± 14.4	63.9 ± 14.5	65.5 ± 14.4	69.5 ± 11.9	< 0.001
Gender, n (%)						< 0.001
Male	1249 (47.3)	244 (37.2)	279 (42.1)	306 (48.6)	420 (60.9)	
Female	1390 (52.7)	412 (62.8)	384 (57.9)	324 (51.4)	270 (39.1)	
Race/ethnicity, n (%)						< 0.001
Non-Hispanic White	1852 (70.2)	394 (60.1)	465 (70.1)	446 (70.8)	547 (79.3)	
Non-Hispanic Black	358 (13.6)	140 (21.3)	82 (12.4)	70 (11.1)	66 (9.6)	
Mexican American	167 (6.3)	44 (6.7)	48 (7.2)	46 (7.3)	29 (4.2)	
Others	262 (9.9)	78 (11.9)	68 (10.3)	68 (10.8)	48 (7)	
Marital status, n (%)						0.732
Married or living with partners	1037 (39.3)	255 (38.9)	263 (39.7)	238 (37.8)	281 (40.7)	
Living alone	1602 (60.7)	401 (61.1)	400 (60.3)	392 (62.2)	409 (59.3)	
Education 1evel(year), n (%)						0.548
<9	529 (20.0)	124 (18.9)	137 (20.7)	128 (20.3)	140 (20.3)	
9-12	586 (22.2)	145 (22.1)	130 (19.6)	147 (23.3)	164 (23.8)	
>12	1524 (57.7)	387 (59)	396 (59.7)	355 (56.3)	386 (55.9)	
Family income, n (%)						0.003
Low	622 (23.6)	173 (26.4)	154 (23.2)	143 (22.7)	152 (22)	
Medium	1073 (40.7)	231 (35.2)	260 (39.2)	261 (41.4)	321 (46.5)	
High	944 (35.8)	252 (38.4)	249 (37.6)	226 (35.9)	217 (31.4)	
Smoking status, n (%)						< 0.001
Never	1182 (44.8)	327 (49.8)	304 (45.9)	284 (45.1)	267 (38.7)	
Current	1038 (39.3)	220 (33.5)	248 (37.4)	254 (40.3)	316 (45.8)	
Former	419 (15.9)	109 (16.6)	111 (16.7)	92 (14.6)	107 (15.5)	
Alcohol drinking status, n (%)						0.042
Never	339 (12.8)	91 (13.9)	86 (13)	88 (14)	74 (10.7)	
Former	585 (22.2)	130 (19.8)	141 (21.3)	135 (21.4)	179 (25.9)	
Mild	1134 (43.0)	271 (41.3)	282 (42.5)	266 (42.2)	315 (45.7)	
Moderate	327 (12.4)	97 (14.8)	86 (13)	77 (12.2)	67 (9.7)	
Heavy	254 ( 9.6)	67 (10.2)	68 (10.3)	64 (10.2)	55 (8)	
Body mass index (kg/m^2^), Mean (SD)	29.2 ± 6.5	29.0 ± 6.3	29.4 ± 6.6	29.4 ± 6.5	28.9 ± 6.6	0.37
Cardiovascular disease, n (%)	646 (24.5)	133 (20.3)	148 (22.3)	161 (25.6)	204 (29.6)	< 0.001
Hypertension, n (%)	1686 (63.9)	390 (59.5)	410 (61.8)	394 (62.5)	492 (71.3)	< 0.001
Diabetes, n (%)	720 (27.3)	149 (22.7)	181 (27.3)	180 (28.6)	210 (30.4)	0.012
Hemoglobin(g/L), Mean ± SD	13.9 ± 1.5	13.8 ± 1.4	13.9 ± 1.4	13.9 ± 1.5	13.9 ± 1.6	0.081
Platelet(10^9^/L), Mean ± SD	237.1 ± 69.7	237.5 ± 67.8	236.5 ± 67.4	242.1 ± 72.9	232.9 ± 70.6	0.121
Alanine transaminase (IU/L), Median (IQR)	20.0 (16.0, 26.0)	20.0 (16.0, 27.0)	20.0 (16.0, 26.0)	19.0 (16.0, 25.0)	19.0 (15.0, 25.0)	0.048
Albumin(g/L), Mean ± SD	41.7 ± 3.3	41.8 ± 3.2	42.0 ± 3.2	41.8 ± 3.2	41.2 ± 3.5	< 0.001
Creatinine (μmol/L), Median (IQR)	81.3 (67.2, 97.2)	78.7 (65.2, 92.8)	79.6 (65.4, 92.8)	79.6 (66.3, 97.2)	86.6 (71.6, 106.1)	< 0.001
Lactate dehydrogenase (IU/L), Mean ± SD	138.6 ± 33.3	139.0 ± 33.1	136.2 ± 28.1	138.0 ± 35.6	141.2 ± 35.6	0.045
All-cause mortality n (%)	713 (27.0)	125 (19.1)	150 (22.6)	176 (27.9)	262 (38)	< 0.001
Cardiovascular mortality, n (%)	149 (5.6)	20 (3)	24 (3.6)	36 (5.7)	69 (10)	< 0.001
Time(months), Median (IQR)	77.0 (45.0, 121.0)	80.0 (45.0, 128.0)	78.0 (48.0, 123.0)	82.0 (47.0, 122.0)	68.5 (38.0, 108.8)	< 0.001

NLR, neutrophil-to-lymphocyte ratio; SD, standard deviation; IQR, interquartile range

### Associations between NLR and mortality

**Figure 2:**
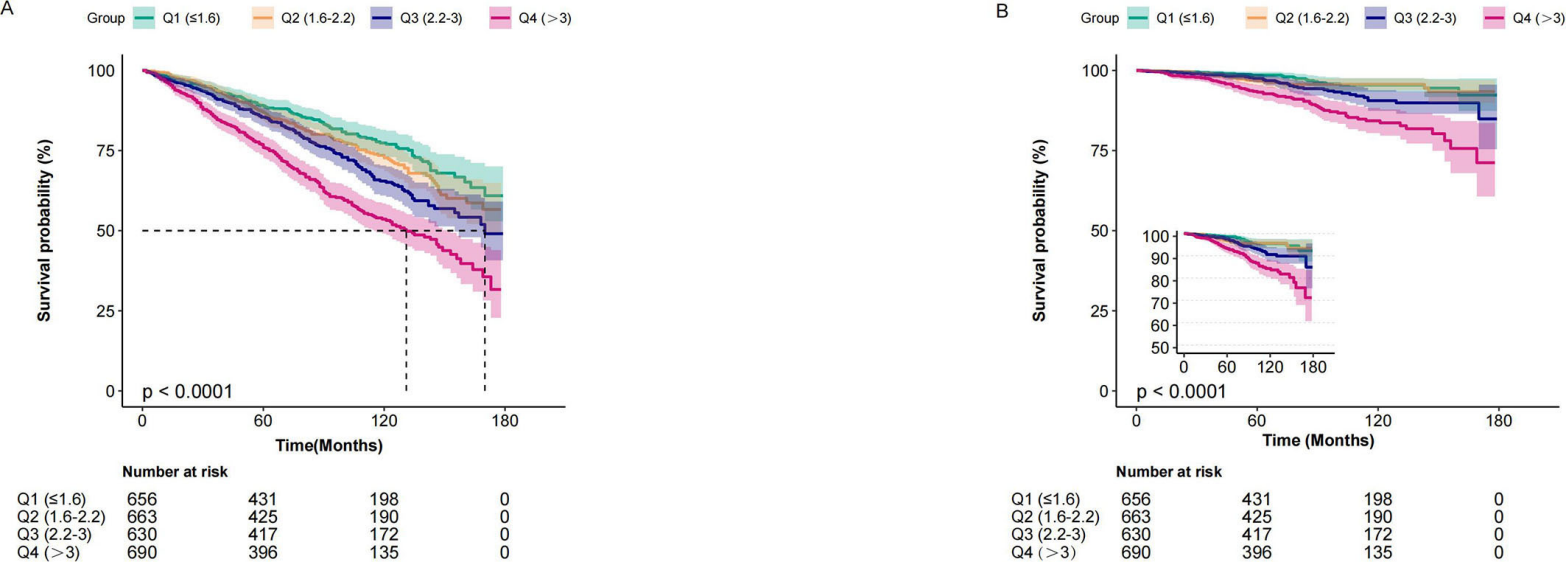
Kaplan–Meier curves of the survival rate and the prevalence (%) of cancer patients with varying NLR. **(A)** all-cause mortality. **(B)** cardiovascular mortality.

**Table 2** presents the results of the multivariable Cox regression analysis that examined the association between the NLR, all-cause mortality, and CVD mortality. A high NLR as a continuous variable was associated with an increased all-cause mortality (HR, 1.09; 95% CI, 1.05-1.12; p <0.001) and CVD mortality (HR, 1.12; 95% CI, (1.05~1.19) 1.05-1.12; p <0.001) after adjusting for age, sex, marital status, race/ethnicity, educational level, PIR, BMI, smoking status, alcohol drinking status, hypertension, diabetes, CVD, hemoglobin, platelet, ALT, creatinine, albumin and LDH. When NLR was evaluated as a categorical variable, the adjusted HR values for NLR and all-cause mortality in Q2 (1.6-2.2), Q3 (2.2-3), and Q4 (>3) were 1.06 (95% CI: 0.83-1.34, p = 0.062), 1.12 (95% CI: 0.89-1.42, p = 0.334), and 1.3 (95% CI: 1.04-1.63, p = 0.021), respectively, compared with individuals with lower Q1 (≤1.6) NLR. Meanwhile, the adjusted HR values for NLR and CVD mortality in Q2, Q3, and Q4 were 0.92 (95% CI: 0.5-1.69, p = 0.062), 1.24 (95% CI: 0.71-12.19, p = 0.334), and 1.76 (95% CI: 1.04-2.97, p = 0.034), respectively, compared with individuals with lower NLR Q1 (≤1.6). These findings suggest a potential association between higher NLR categories and increased risk of all-cause and CVD mortality. Kaplan-Meier analysis revealed that the survival rate of the group with an elevated NLR was significantly lower than that of the group with a lower NLR for both all-cause and CVD mortality (both p < 0.0001) (**Figure 2**).

**Table 2:** Association between NLR and mortality in adults with cancer.

Characteristic	Crude model		Model 1		Model 2		Model 3	
	HR (95%CI)	*p*-Value	HR (95%CI)	*p*-Value	HR (95%CI)	*p*-Value	HR (95%CI)	*p*-Value
All-cause mortality								
NLR	1.18 (1.15~1.21)	<0.001	1.12 (1.08~1.16)	<0.001	1.11 (1.08~1.15)	<0.001	1.09 (1.05~1.12)	<0.001
NLR category								
Q1(≤1.6)	1(Ref)		1(Ref)		1(Ref)		1(Ref)	
Q2(1.6-2.2)	1.21 (0.95~1.53)	0.122	1.05 (0.83~1.33)	0.691	1.03 (0.81~1.31)	0.818	1.06 (0.83~1.34)	0.659
Q3(2.2-3)	1.5 (1.19~1.89)	<0.001	1.19 (0.94~1.5)	0.15	1.16 (0.92~1.46)	0.223	1.12 (0.89~1.42)	0.334
Q4(>3)	2.36 (1.9~2.92)	<0.001	1.43 (1.15~1.78)	0.002	1.41 (1.13~1.77)	0.002	1.3 (1.04~1.63)	0.021
Cardiovascular mortality								
NLR	1.21 (1.16~1.27)	<0.001	1.14 (1.08~1.21)	<0.001	1.14 (1.08~1.21)	<0.001	1.12 (1.05~1.19)	<0.001
NLR category								
Q1(≤1.6)	1(Ref)		1(Ref)		1(Ref)		1(Ref)	
Q2(1.6-2.2)	1.2 (0.67~2.18)	0.539	0.98 (0.54~1.79)	0.958	0.92 (0.5~1.7)	0.8	0.92 (0.5~1.69)	0.784
Q3(2.2-3)	1.91 (1.11~3.3)	0.02	1.39 (0.8~2.41)	0.247	1.32 (0.75~2.32)	0.331	1.24 (0.71~2.19)	0.451
Q4(>3)	3.87 (2.35~6.37)	<0.001	1.93 (1.15~3.22)	0.012	1.94 (1.15~3.27)	0.013	1.76 (1.04~2.97)	0.034

NLR, neutrophil-lymphocyte ratio; Q, quantiles; HR, Hazard Ratio; CI, Confidence Interval; Ref, reference; Model1: Adjusted for variables (age, sex, race, marital status, poverty income ratio, and education); Model2: Adjusted for Model1 and smoke, alcohol drinking status, body mass index (BMI), hypertension, diabetes, and cardiovascular disease; Model3: Adjusted for Model2 and hemoglobin, platelet, alanine aminotransferase, creatinine, albumin, and lactate dehydrogenase levels.

### Subgroup analysis

**Figure 3:**
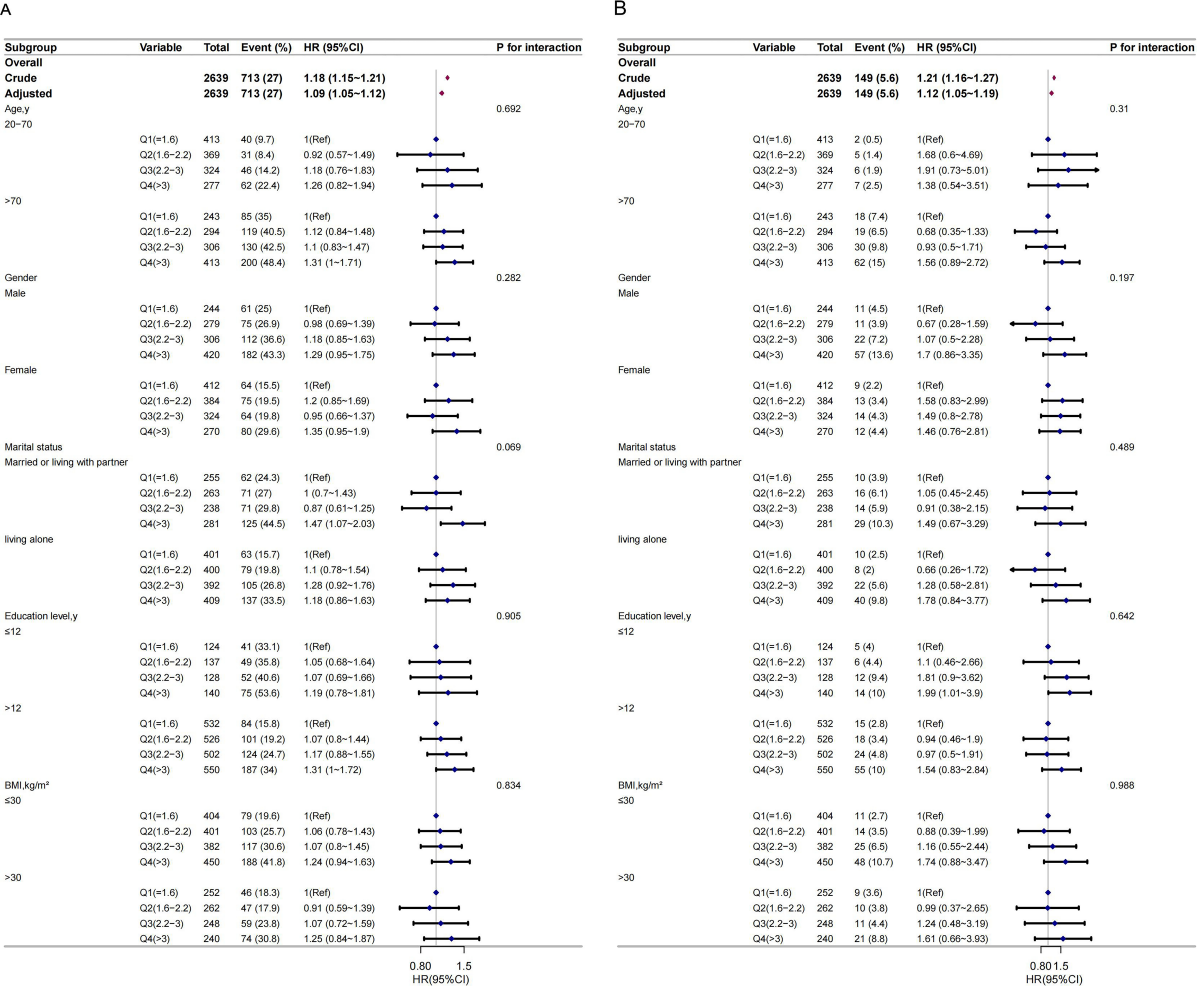
The relationship between NLR and all-cause mortality **(A)** and cardiovascular mortality **(B)** according to basic features. Except for the stratification component itself, each stratification factor was adjusted for all other variables (age, sex, race, marry, poverty income ratio (PIR), education, smoke, alcohol drinking status, body mass index (BMI), hypertension, diabetes, cardiovascular disease (CVD), hemoglobin, platelet, creatinine, alanine aminotransferase (ALT), albumin, and lactate dehydrogenase levels (LDH).

Subgroup analysis was performed to evaluate possible effect modifications in the association between the NLR and all-cause and CVD mortality. These findings were robust even after considering various factors. No significant interactions were observed in any of the subgroups even after stratification by age, sex, marital status, educational level, or BMI (**Figure 3**).

### Applying RCS analysis

**Figure 4:**
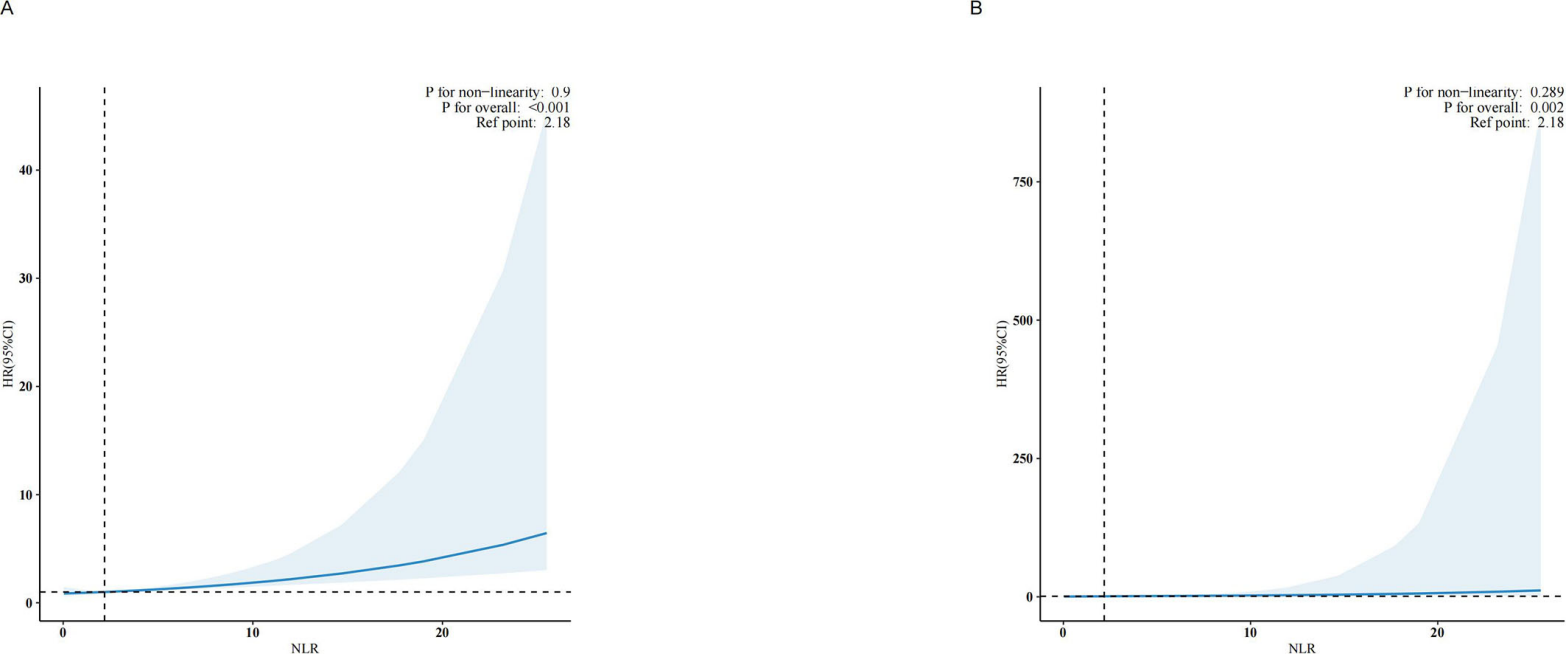
Association between NLR and all-cause mortality **(A)** and cardiovascular mortality **(B)** hazard ratio. Solid and dashed lines represent the predicted value and 95% confidence intervals. They were adjusted for age, sex, race, marry, poverty income ratio (PIR), education, smoke, alcohol drinking status, body mass index (BMI), hypertension, diabetes, cardiovascular disease (CVD), hemoglobin, platelet, creatinine, alanine aminotransferase (ALT), albumin, and lactate dehydrogenase levels (LDH).

Analysis using RCS suggested a linear relationship between NLR and all-cause (**Figure 4A**, p for nonlinearity = 0.9) and CVD mortality (**Figure 4B**, p for nonlinearity = 0.289). The association between NLR and mortality demonstrated an increasing trend as NLR increased, suggesting a possible correlation between an elevated NLR and an increased risk of mortality, as depicted in **Figure 4**.

### Sensitivity analysis

We conducted an analysis comparing NLR Q1-3 versus Q4 using a cutoff value of 3, and also performed a corresponding analysis with a cutoff value of 5. The adjusted hazard ratios (HR) for NLR and all-cause mortality, as well as CVD mortality, in NLR Q2 (>3) were 1.25 (95% CI: 1.06-1.46, p = 0.007) and 1.68 (95% CI: 1.19-2.35, p = 0.003), respectively, compared to individuals with lower NLR (Q1 ≤ 3) (see **Supplementary Table S2**). For individuals with NLR Q2 (>5), the adjusted HR values for all-cause mortality and CVD mortality were 1.78 (95% CI: 1.37-2.31, p < 0.001) and 3.04 (95% CI: 1.87-4.94, p < 0.001), respectively, compared to those with lower NLR (Q1 ≤ 5) (see **Supplementary Table S3**).

## Discussion

Our extensive research, involving a large and nationally representative cohort of adults in the US, revealed that a higher NLR was independently correlated with an increased risk of all-cause and CVD mortality in adults with cancer. These findings were robust even after considering various factors and no significant interactions were found in the subgroup analysis. Kaplan-Meier analysis revealed lower survival rates among individuals with higher NLR values for both all-cause and CVD mortality. Furthermore, analysis using RCS indicated a positive linear relationship between NLR levels and both all-cause and CVD mortality. This underscores the potential value of the NLR as an affordable and easily accessible marker for stratifying cancer risk and predicting prognoses in clinical practice.

Heart disease and cancer are leading causes of death (8). In 2015, 17.7 million deaths worldwide were due to CVD and 8.8 million were due to cancer (8). Cancer survivors have a higher risk of CVD because of shared lifestyle factors and cancer treatment toxicities (18, 19). With advancements in cancer care, the number of cancer survivors has increased (19). It is important to focus on long-term cardiovascular health through lifestyle changes and monitoring for potential treatment-related heart issues. Collaboration between oncology and cardiology professionals is crucial for providing comprehensive care. Our study found that a higher NLR was proportionally related to an increased risk of incident all-cause and CVD mortality among individuals with cancer from the NHANES dataset spanning 2005 to 2018. These findings were consistent with those of other observational studies. A recent meta-analysis study has corroborated the association between a high NLR and unfavorable overall survival in numerous solid tumors, including gynecologic cancers, colorectal cancer, breast cancer, non-small cell lung cancer, testicular cancer, ovarian cancer and colon cancer (20–29). Previous studies have investigated the correlation between NLR and all-cause and CVD mortality (15, 30, 31). For instance, Gai ying Dong et al. found that individuals with diabetes and a higher NLR had a significantly increased risk of all-cause and CVD mortality (15). Moreover, a recent prospective longitudinal cohort study involving patients with hypertension demonstrated a notable positive association between the NLR and all-cause and CVD mortality (30). Furthermore, Er ye Zhou found that a high NLR was independently associated with increased long-term mortality risk in American adults diagnosed with rheumatoid arthritis (31). NLR is an easily accessible and cost-effective biomarker, and its integration into established prognostic scores for clinical decision-making requires further exploration.

Research among childhood and young adult cancer survivors (diagnosed under 40 years of age) has revealed a significantly elevated risk of CVD compared with the general population. This heightened risk is primarily attributed to exposure to cardiotoxic treatments such as anthracyclines and chest radiation during early life, coupled with the subsequent development of new cardiovascular risk factors (CVRFs, diabetes, hypertension, and dyslipidemia) with age (32, 33). However, the extent of CVD risk in individuals diagnosed with cancer at an older age (40 years and above) is less well understood. This demographic represents 95% of all new cancer diagnoses in the United States and is characterized by a high prevalence of CVRFs (34, 35). NLR has demonstrated its utility as a significant prognostic biomarker, correlating with the clinical outcomes of CVD. It correlates with both the severity and mortality of conditions such as acute coronary syndrome, coronary artery disease, and heart failure, underscoring its predictive capabilities in the context of cardiovascular health, as detailed in the literature (36, 37). Furthermore, increased peripheral NLR has been identified as a negative prognostic indicator of various types of cancer (9, 13, 21–23, 25, 27, 38). The precise mechanisms underlying the link between high NLR and poor outcomes in patients with cancer remain poorly understood. One potential reason for the prognostic importance of the NLR is its association with inflammation. Elevated levels of neutrophils, which indicate an inflammatory response, suppress the immune system by reducing the cytolytic activity of immune cells, such as activated T cells, natural killer cells, and lymphocytes (39). The significance of lymphocytes has been underscored in numerous studies showing that increased tumor infiltration by lymphocytes is linked to improved responses to cytotoxic treatment and a better prognosis in cancer patients (40). Tumors and their associated host cells such as leukocytes produce inflammatory cytokines and chemokines that contribute to malignant progression (41). An elevated NLR has been linked to increased peritumoral macrophage infiltration and increased levels of interleukin (IL) 17 (42). Neutrophils, along with other cell types, such as macrophages, are known to secrete factors that promote tumor growth. These include hepatocyte growth factor, vascular endothelial growth factor, matrix metalloproteinases, IL-6, IL-8, and elastases (43–47), which together help create a supportive microenvironment. These components are included in the Glasgow Prognostic Score, which has been shown to predict prognosis in various types of solid tumors (12).

This study has several notable strengths, including its large sample size, population-based design, and ability to investigate the relationship between NLR and both all-cause and CVD mortality, as well as various subgroups simultaneously. This study utilized a territory-wide, thoroughly validated electronic healthcare database that contains comprehensive records of diagnoses, hospitalizations, and drug-dispensing details. This robust dataset enabled the collection of the pertinent information necessary to mitigate common biases encountered in conventional observational studies, such as selection and recall biases.

Despite these strengths, it is essential to acknowledge the limitations of this study. First, the cross-sectional and observational nature of the analysis restricts the ability to establish definitive causal relationships regarding the interplay between the NLR and all-cause and CVD. Second, this study is the conservative nature of the Bonferroni correction, which may increase the risk of type II errors. Moreover, the subgroup analyses are exploratory and subject to uncertainty, requiring further validation in larger and more diverse populations. Third, the study is limited by the lack of detailed data on cancer staging, treatment methods (e.g., surgery, chemotherapy, radiation), performance status (PS), and patient diagnosis status (new or recurrent). Future studies should incorporate these variables to enhance cancer outcome assessments. Lastly, NLR was measured based on survey values rather than at cancer diagnosis. As a dynamic variable, NLR can change before and after treatment, so its prognostic value may vary with cancer stage. Future studies should assess NLR at multiple time points for a more accurate evaluation of its significance. Nevertheless, the data presented in this study effectively explored the associations between the NLR and all-cause and CVD mortality outcomes, contributed additional evidence to the existing literature, and revealed variations across continents and ethnicities.

## Conclusion

In this study, our findings suggest that elevated levels of the NLR may be associated with increased all-cause and CVD mortality. These results are significant and warrant further investigation. This association could be crucial for clinicians to consider when managing patients with cancer who are at risk for all-cause and CVD mortality.

## Data Availability

The datasets presented in this study can be found in online repositories. The names of the repository/repositories and accession number(s) can be found below: All the datasets are available on the NHANES website (https://www.cdc.gov/nchs/nhanes/?CDC_AAref_Val=https://www.cdc.gov/nchs/nhanes/index.htm).
