# Supplementary material for: Association between neutrophil-to-lymphocyte ratio and all-cause and cardiovascular mortality among adults with cancer from NHANES 2005-2018: a retrospective cohort study
Source: Front Oncol. 2025 Mar 18;15:1521099. doi: 10.3389/fonc.2025.1521099 (PMC11959702; doi:10.3389/fonc.2025.1521099)
Supplement: Supplementary file 1 [file Table1.docx]

**Supplementary Table 1.** Specific cancer Types and Numbers of Cancers

| **Code** | **Variable Name:**  **What kind of cancer was it?** | **Numbers** |
| --- | --- | --- |
| 10 | Bladder | 67 |
| 11 | Blood | 6 |
| 12 | Bone | 9 |
| 13 | Brain | 13 |
| 14 | Breast | 399 |
| 15 | Cervix(cervical) | 188 |
| 16 | Colon | 155 |
| 17 | Esophagus | 12 |
| 18 | Gallbladder | 1 |
| 19 | Kidney | 45 |
| 20 | Larynx/windpipe | 8 |
| 21 | Leukemia | 26 |
| 22 | Liver | 10 |
| 23 | Lung | 53 |
| 24 | Lymphoma/Hodgkin's | 55 |
| 25 | Melanoma | 160 |
| 26 | Mouth/tongue/lip | 17 |
| 27 | Nervous system | 1 |
| 28 | Ovary(ovarian) | 55 |
| 29 | Pancreas(pancreatic) | 4 |
| 30 | Prostate | 400 |
| 31 | Rectum(rectal) | 8 |
| 32 | Skin(non-melanoma) | 429 |
| 33 | Skin (don't know what kind) | 204 |
| 34 | Soft tissue (muscle or fat)） | 3 |
| 35 | Stomach | 13 |
| 36 | Testis(testicular) | 13 |
| 37 | Thyroid | 52 |
| 38 | Uterus(uterine) | 100 |
| 39 | Other | 115 |
| 99 | Don't know | 18 |
