# Supplementary material for: Association between neutrophil-to-lymphocyte ratio and all-cause and cardiovascular mortality among adults with cancer from NHANES 2005-2018: a retrospective cohort study
Source: Front Oncol. 2025 Mar 18;15:1521099. doi: 10.3389/fonc.2025.1521099 (PMC11959702; doi:10.3389/fonc.2025.1521099)
Supplement: Supplementary file 2 [file Table2.docx]

**Supplementary Table 2.** Association between NLR and mortality in adults with cancer

| Characteristic | Crude model | |  | Model 1 | |  | Model 2 | |  | Model 3 | |
| --- | --- | --- | --- | --- | --- | --- | --- | --- | --- | --- | --- |
|  | HR (95%CI) | *p*-Value |  | HR (95%CI) | *p*-Value |  | HR (95%CI) | *p*-Value |  | HR (95%CI) | *p*-Value |
| All-cause mortality |  |  |  |  |  |  |  |  |  |  |  |
| NLR ≤3 | 1(Ref) |  |  | 1(Ref) |  |  | 1(Ref) |  |  | 1(Ref) |  |
| NLR＞3 | 1.94  (1.66~2.25) | <0.001 |  | 1.33  (1.14~1.56) | <0.001 |  | 1.35  (1.15~1.58) | <0.001 |  | 1.25 (1.06~1.46) | 0.007 |
| Cardiovascular mortality |  |  |  |  |  |  |  |  |  |  |  |
| NLR ≤3 | 1(Ref) |  |  | 1(Ref) |  |  | 1(Ref) |  |  | 1(Ref) |  |
| NLR＞3 | 2.86  (2.07~3.95) | <0.001 |  | 1.71  (1.23~2.38) | 0.002 |  | 1.8  (1.29~2.52) | 0.001 |  | 1.68 (1.19~2.35) | 0.003 |

NLR, neutrophil-lymphocyte ratio; Q, quantiles; HR, Hazard Ratio; CI, Confidence Interval; Ref, reference; Model1: Adjusted for variables (age, sex, race, marital status, poverty income ratio, and education); Model2: Adjusted for Model1 and smoke, alcohol drinking status, body mass index (BMI), hypertension, diabetes, and cardiovascular disease; Model3: Adjusted for Model2 and hemoglobin, platelet, alanine aminotransferase, creatinine, albumin, and lactate dehydrogenase levels.
